# Deep learning-based IDH1 gene mutation prediction using histopathological imaging and clinical data

**DOI:** 10.1016/j.compbiomed.2024.108902

**Published:** 2024-07-21

**Authors:** Riku Nakagaki, Shyam Sundar Debsarkar, Hiroharu Kawanaka, Bruce J. Aronow, V.B. Surya Prasath

**Affiliations:** a Graduate School of Engineering, Mie University, 1577 Kurima-machiya, Tsu, Mie 514-8507, Japan; b Department of Computer Science, University of Cincinnati, OH 45221, USA; c Division of Biomedical Informatics, Cincinnati Children’s Hospital Medical Center, Cincinnati, OH 45229, USA; d Department of Pediatrics, University of Cincinnati, OH 45267, USA; e Department of Biomedical Informatics, College of Medicine, University of Cincinnati, OH 45267, USA

**Keywords:** Digital pathology, Gene mutation, Brain glioma, Weakly supervised, Fusion model, Deep learning, Clinical data

## Abstract

In the field of histopathology, many studies on the classification of whole slide images (WSIs) using artificial intelligence (AI) technology have been reported. We have studied the disease progression assessment of glioma. Adult-type diffuse gliomas, a type of brain tumor, are classified into astrocytoma, oligodendroglioma, and glioblastoma. Astrocytoma and oligodendroglioma are also called low grade glioma (LGG), and glioblastoma is also called glioblastoma multiforme (GBM). LGG patients frequently have isocitrate dehydrogenase (IDH) mutations. Patients with IDH mutations have been reported to have a better prognosis than patients without IDH mutations. Therefore, IDH mutations are an essential indicator for the classification of glioma. That is why we focused on the IDH1 mutation. In this paper, we aimed to classify the presence or absence of the IDH1 mutation using WSIs and clinical data of glioma patients. Ensemble learning between the WSIs model and the clinical data model is used to classify the presence or absence of IDH1 mutation. By using slide level labels, we combined patch-based imaging information from hematoxylin and eosin (H & E) stained WSIs, along with clinical data using deep image feature extraction and machine learning classifier for predicting IDH1 gene mutation prediction versus wild-type across cohort of 546 patients. We experimented with different deep learning (DL) models including attention-based multiple instance learning (ABMIL) models on imaging data along with gradient boosting machine (LightGBM) for the clinical variables. Further, we used hyperparameter optimization to find the best overall model in terms of classification accuracy. We obtained the highest area under the curve (AUC) of 0.823 for WSIs, 0.782 for clinical data, and 0.852 for ensemble results using MaxViT and LightGBM combination, respectively. Our experimental results indicate that the overall accuracy of the AI models can be improved by using both clinical data and images.

## 1. Introduction

In the field of histopathology, pathologists diagnose disease by observing pathological specimens obtained by biopsy and stained with hematoxylin and eosin (H&E) under a microscope. Pathological diagnosis, also known as “final diagnosis” or “definite diagnosis”, is essential for disease detection and classification. Recently, in addition to phenotypic testing, results of genetic testing such as immunohistochemistry (IHC), fluorescence in situ hybridization (FISH), and next-generation sequencing (NGS) have become increasingly important in cancer diagnosis [1–3]. The diagnosis with histology and molecular information is called integrated diagnosis. However, these genetic tests have some problems, such as high medical costs, a turnaround time of several days to two weeks for results, and the need for specialized medical facilities and health institutions to perform the tests.

Traditionally radiological magnetic resonance (MR) images are used in predicting gene mutations in brain gliomas [4–6]. On the other hand, there has been increased research on cancer detection, classification, and gene mutation prediction in pathological imaging using artificial intelligence (AI) technology [7–12]. Furthermore, studies have reported the prediction of gene mutations using deep learning from pathological images [13,14]. However, these studies only used pathology images, which means they might not accurately predict cases where gene mutations do not appear in the images. Therefore, there are certain limitations to accurately predicting gene mutations from the phenotype of pathological images. For this reason, in this work, we propose a method to predict gene mutations by combining pathological images and clinical data. Even if histological features caused by gene mutations do not manifest via H & E pathology imaging data, augmenting appropriate clinical metadata is expected to help improve the overall predictions. Further, this approach can lead to more efficient diagnosis of gliomas and reduce patient medical costs by identifying gene mutations before going for costly genetic testing.

According to the 2021 WHO classification of brain tumors [3], Adult-type diffuse gliomas are classified into three types as follows:

1. Astrocytoma, IDH-mutant,
2. Oligodendroglioma, IDH-mutant, and 1p/19q-codeleted,
3. Glioblastoma, IDH-wildtype.

**Fig. 1.**
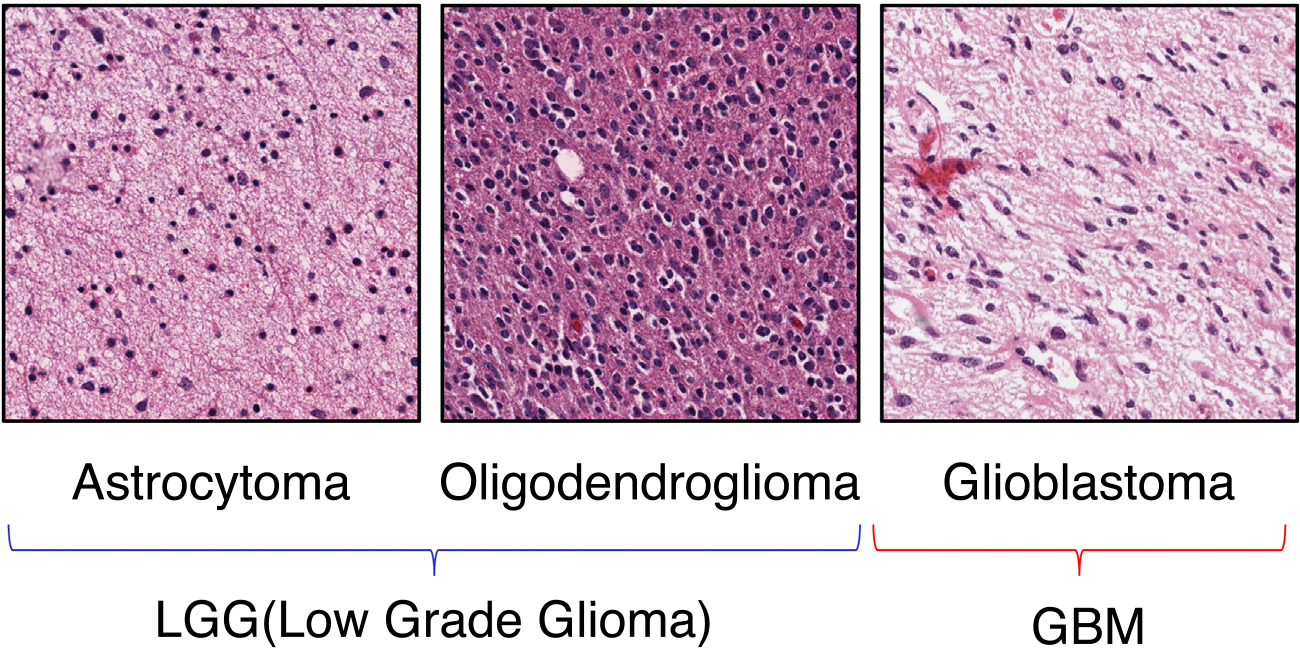
Example of HE-stained images of gliomas. Astrocytoma and oligodendroglioma are also called low-grade glioma (LGG). Glioblastoma is also known as glioblastoma multiforme or GBM. It is the most aggressive type of glioma. Histological findings differ depending on the type.

Fig. 1 shows an example of pathological images of glioma. Astrocytoma and oligodendroglioma are usually called LGG (Low-Grade Glioma), and glioblastoma is called GBM. GBM is grade 4 and the highest grade of glioma [15]. In general, the 5-year survival rate of LGG patients is better than GBM patients [16]. IDH mutations are frequently found in LGG patients. Patients with IDH mutations have been reported to have a better prognosis than those without IDH mutations [17,18]. Therefore, IDH mutations are helpful indicators for the classification of gliomas.

In pathological diagnosis, genotypic analysis is performed along with phenotypic analysis. If gene mutations can be identified without genetic testing, it will improve the efficiency of diagnosis and reduce the cost of treatment for patients. This paper focuses on the IDH1 mutation, the most common mutation among IDH mutations in LGG patients. This work aims to classify the presence/absence of IDH1 mutation by combining pathological images and clinical data. We use large-scale histopathology H & E imaging and associated data from glioma patients [19]. We utilize a weakly supervised deep learning model and clinical data encoded with a LightGBM machine learning classifier [20,21] to obtain IDH1 mutation versus wild-type. Our ensemble AI approach obtains accurate prediction in the fusion approach and is further amenable to interpretable features thereby resulting in explainable AI (xAI) models.

We organized the paper as follows. Section 2 introduces our overall fusion method combining imaging and clinical data. Section 3 provides detailed experimental results on a large-scale dataset of 546 patients. Finally, Section 4 provides the conclusions and future works.

## 2. Materials and methods

### 2.1. The Cancer Genome Atlas (TCGA) dataset

**Fig. 2.**
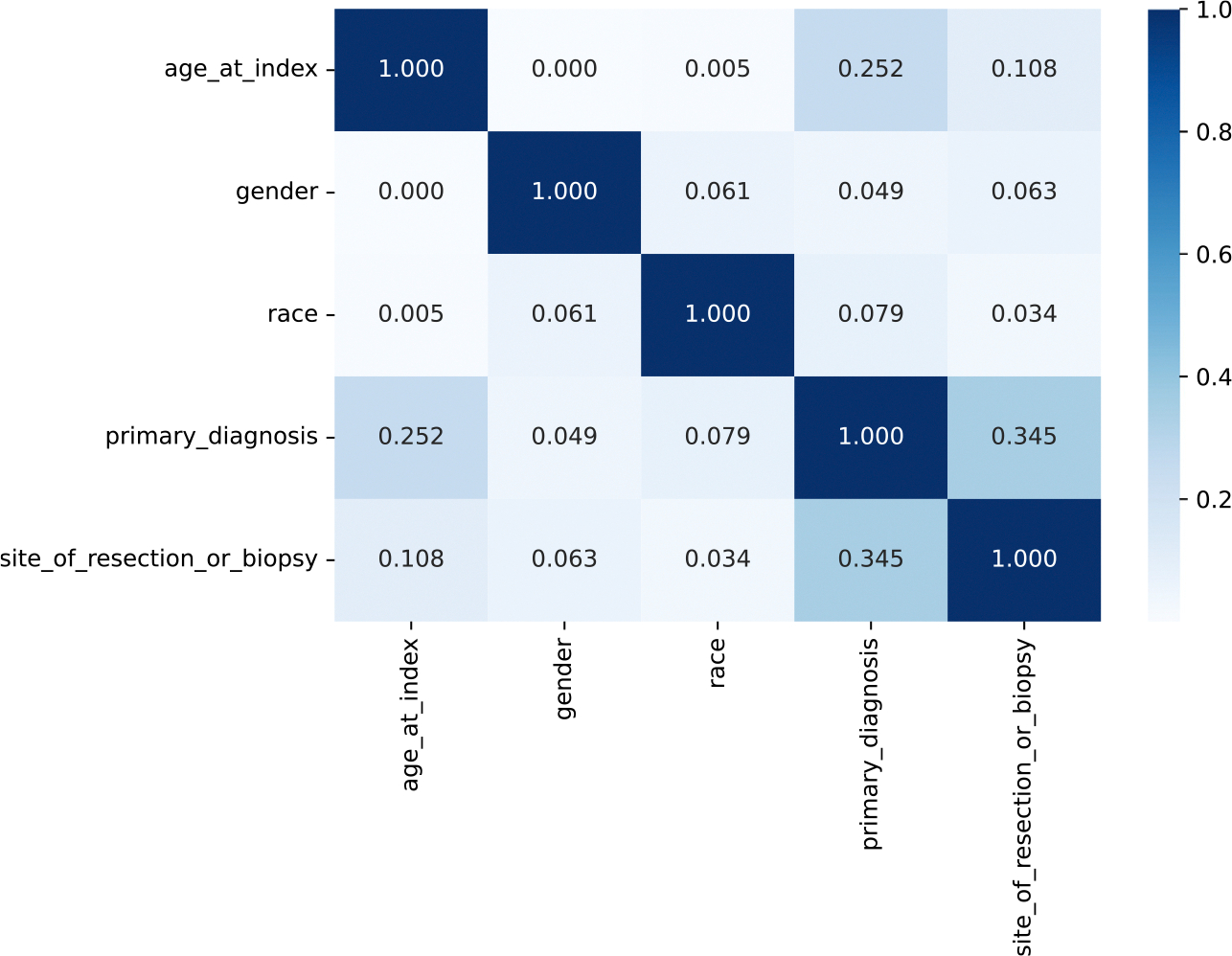
Correlation matrix. Numerical–Categorical: Correlation ratio, Categorical–Categorical: Cramer’s V.

In this work, we used Whole-Slide Images (WSIs), gene mutation data, and clinical data for gliomas obtained from the NCI Genomic Data Commons (GDC) [22]. We labeled the WSIs and clinical data with the presence or absence of IDH1 mutation (IDH1 or Wild-Type). Table 1 shows the summary of the dataset, and we chose five non-redundant clinical variables and we omitted the variables that have too many missing values. *N* is the number of patients. We collected data from 321 patients with IDH1 mutation and 225 patients with Wild-Type and used 603 WSIs for each label. Only age is a numerical variable. All variables except age are categorical variables. Fig. 2 shows the correlation matrix of the variables. The correlations between age and categorical variables were calculated as the correlation ratio, and the associations between categorical variables were calculated as the Cramer’s V.

### 2.2. Proposed approach

**Fig. 3.**
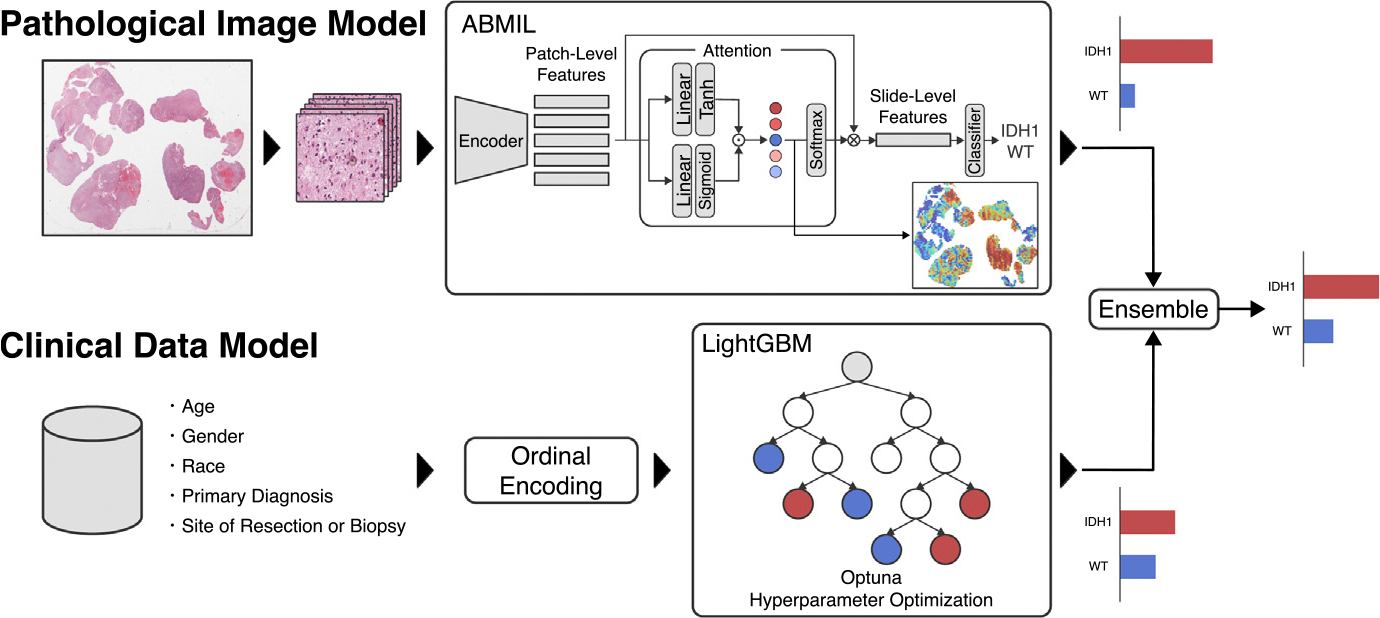
Overview of the ensemble method. In the WSI-based approach, a weakly supervised learning method called ABMIL performs slide-level classification. In the clinical data-based approach, classification is performed using LightGBM. The average confidence of both approaches is calculated and used as the final output.

Fig. 3 shows the overall proposed approach of this work. We trained separate deep and machine learning models on image and clinical data. The final prediction results were obtained by ensembling their outputs. The classification performances were evaluated by 10-fold Monte Carlo cross-validation. The dataset was randomly divided into a training set (80%), a validation set (10%), and a test set (10%) for each fold. If one patient had multiple WSIs, they were carefully divided so that all of them would be in the same set.

### 2.3. WSI-based classification

**Fig. 4.**
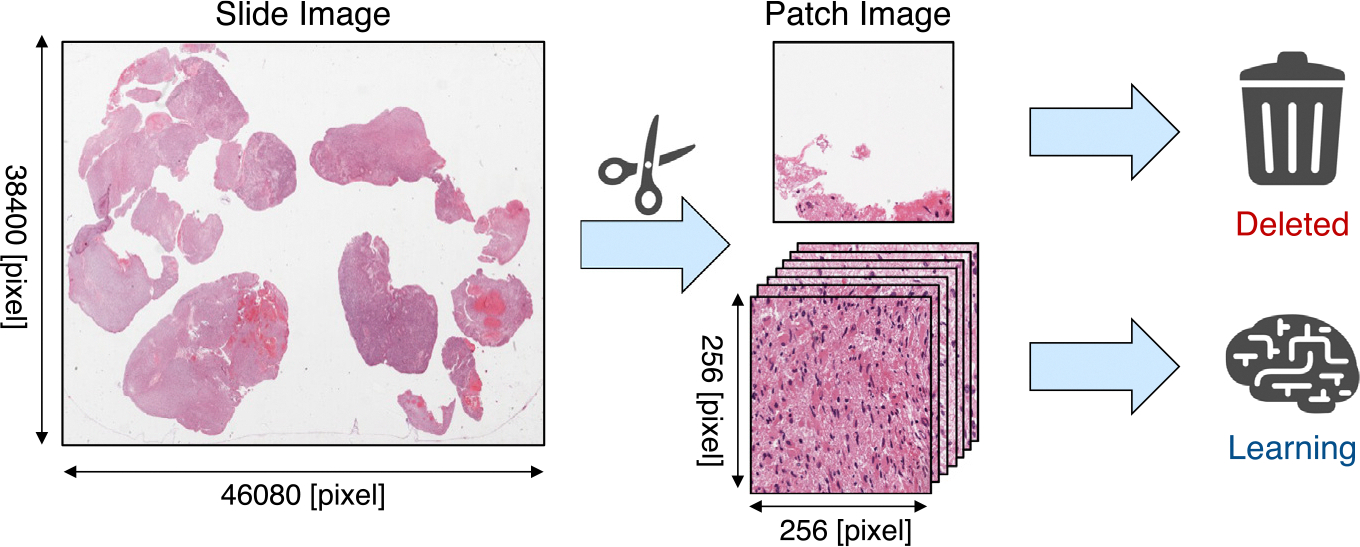
Preprocessing of WSIs for informative patches. We remove patches that contain excessive white spaces.

In preprocessing the images, we divided the WSIs into 256 × 256 pixels patches because the WSIs size was too large to analyze. We removed the white background region and used the region containing sufficient cellular tissue as the dataset for training the image model, see Fig. 4. Pathological images do not generally have uniform features throughout the image. However, our data had no patch-level labels; only slide-level labels were available. Therefore, in this work, we employed the attention-based deep multiple instance learning (ABMIL) [23]. ABMIL is a weakly supervised learning method that classifies only at the slide-level labels without requiring patch-level annotations. Attention-based learning automatically identifies the regions of interest in the WSI. Thus, it is expected that the model will capture histological features caused by IDH1 mutation. First, the patch images are input to the encoder and embedded into patch-level feature vectors of 512–4096 dimensions. Next, each patch-level feature is weighted using the attention mechanism, and the patch-level features are aggregated into slide-level features. This weight is called attention score. The attention scores were also used to visualize an attention heatmap. The following equations represents the gated attention mechanism,

(1) $$z=\sum_{k=1}^{K}a_{k}h_{k},$$

(2) $$a_{k}=\frac{exp\left\{w^{⊤}\left(tanh\left({Vh}_{k}^{⊤}\right)\;⊙\;sigm\left({Uh}_{k}^{⊤}\right)\right)\right\}}{\sum_{j=1}^{K}exp\left\{w^{⊤}\left(tanh\left({Vh}_{j}^{⊤}\right)\;⊙\;sigm\left({Uh}_{j}^{⊤}\right)\right)\right\}},$$

where h is the input vector, w,V, and U are trainable parameters and ⊙ is an element-wise multiplication. Slide-level prediction results are obtained by inputting the features z into the classifier, aggregated from the patch-level to the slide-level by attention mechanism.

In this work, we first compared eight deep learning models to identify the optimal encoder for our purpose. The DL models selected for comparison included convolutional neural network (CNN) models, Transformer models, and hybrid models combining convolutional and Transformer architecture. All these encoders were pre-trained on ImageNet. We used the output of average pooling as feature vectors. The patch images were input to the encoder without data augmentation, such as flipping, rotation, or cropping. However, the CoAtNet was an exception. We resized patch images into 224 × 224 pixels because an error occurred with 256 × 256 pixels image. All models were trained for at least 50 epochs and up to a maximum of 100 epochs, batch size 1. Early stopping is used when validation loss has not decreased continuously by more than 20 epochs. Other hyperparameters, such as learning rate, were optimized by Optuna [24]. Table 2 summarizes the hyperparameters for each encoder. Hyperparameters with high ROC-AUC were adopted and used for ensemble learning.

In addition, we also performed an experiment using Clustering-constrained Attention Multiple Instance Learning (CLAM) [25] for comparison. Note that CLAM’s ResNet50 and our ResNet50 have a different number of parameters because of the different final layers used.

### 2.4. Clinical data-based classification

We used five clinical features, including age, gender, race, primary diagnosis, and site of resection or biopsy. “Site of resection or biopsy” represents the anatomical site of malignant tumors in the patient’s brain, see Table 1. All features were categorical variables except age, so we converted them into numerical values using the Ordinal Encoder. LightGBM was employed as the classification model for the clinical data. LightGBM can handle missing values directly and is a lightweight operation. The hyperparameters of LightGBM were optimized by Optuna. Table 3 summarizes the hyperparameters for LightGBM. In addition, we examined feature importance using SHAP (SHapley Additive exPlanations) [26].

### 2.5. Ensemble learning of WSI and clinical data

After training each model separately, we conducted ensemble learning by taking the average of the probabilities from both models to obtain a final prediction. This is called “Soft Voting”. Soft Voting has the advantage that any classifier can be used. We employed it since the pipeline of this paper is a fusion of a deep learning model and a decision tree model. For example, consider a case where the WSI-based model predicts a probability of IDH1 to be 0.7, while the clinical data-based model predicts a probability of 0.5. Using Soft Voting, the ensemble results would be calculated as (0.7 + 0.5)∕2 = 0.6. This is how we can obtain a final prediction considering pathological images and clinical data.

Ensemble Learning is a method of combining multiple models to achieve higher performance than individual models. It is expected that such a late fusion approach of combining pathological images and clinical data will complement each other and improve the prediction accuracy of gene mutations.

## 3. Experimental results and discussion

### 3.1. Comparisons of WSI feature extractor

We evaluated the classification performance of the WSI-based, the clinical data-based, and the ensemble approaches using 10-fold Monte Carlo cross-validation. For each approach, we calculated accuracy, precision, recall, f1-score, the area under the ROC curve (ROC-AUC), and the area under the precision–recall curve (PR-AUC). Table 4 summarizes the experimental results, including each metric’s mean and standard deviation across the 10-fold cross-validation. The classification performance of each encoder is shown in the WSI row of Table 4. Our results are run on a NVIDIA V100 graphical processing unit (GPU) with 256 RAM. The running time varies between 1 h (for CNN models) to 4 h (for transformer models).

As a result, CoAtNet had the highest scores for many evaluation metrics, achieving an average accuracy of 0.752 over 10-fold cross-validation. In contrast, VGG16 showed the lowest performance. EfficientNet showed performance comparable to other encoders despite having fewer parameters. The AUC is an important metric as it allows for the evaluation of model performance independently of the threshold. Additionally, because our dataset is balanced, not imbalanced, ROC-AUC is the most interesting metric in this work. MaxViT and CoAtNet had ROC-AUCs of 0.823 and 0.821, respectively. These models achieved higher results than 0.820. These models are hybrids of CNN and Vision Transformer architecture. We found their performance to be relatively higher than that of purely convolutional or transformer-based models. Apart from MaxViT and CoAtNet, the other encoders showed almost identical ROC-AUC scores.

### 3.2. Comparisons of model performance with the ensemble of image and clinical data

The classification performance of the clinical data-based and the ensemble approach is shown in Table 4. The LightGBM for clinical data achieved a ROC-AUC of 0.782. The ensemble of WSI and clinical data improved the average performance across all models and all metrics, except for the precision of CoAtNet.

**Fig. 5.**
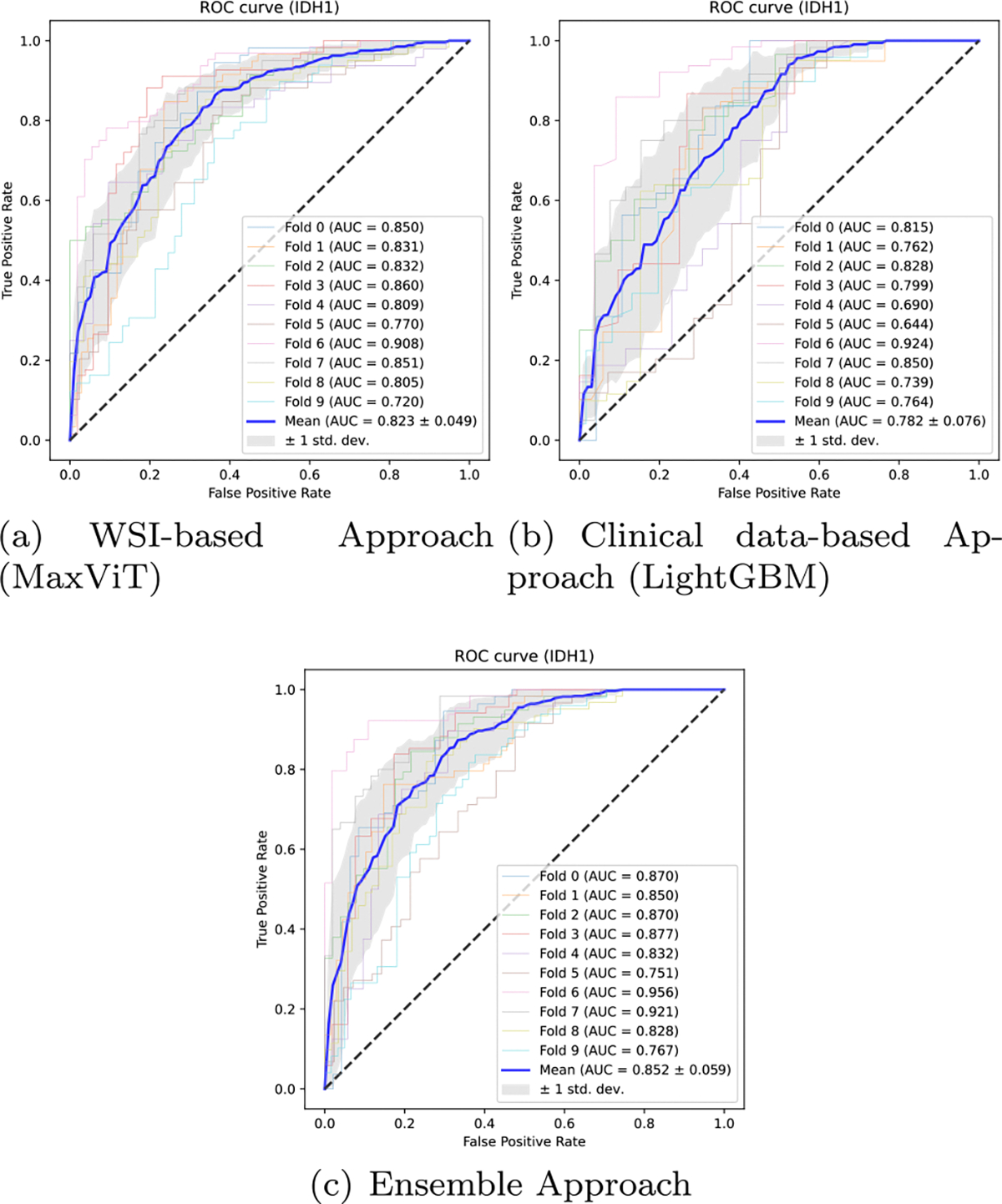
ROC curve. The results are based on (a) WSI, (b) clinical data, and (c) ensemble approaches, respectively. The AUCs for each fold and the average ROC curves are plotted.

Next, we compare the best encoder of WSI and ensemble for each metric. For accuracy and precision, CoAtNet from WSI is lesser than MaxViT from the ensemble. The WSI’s CoAtNet has an accuracy of 0.752, while the ensemble’s MaxViT has an accuracy of 0.778. As for precision, both have an average of 0.765, but MaxViT from the ensemble has a smaller standard deviation. For recall, WSI’s ResNet50(CLAM) is 0.819, and the ensemble’s ResNet50(ABMIL) is 0.859. We also found that the ensemble substantially improved the recall for all encoders. For the F1-score, ResNet50(ABMIL) from the ensemble is also higher than CoAtNet from WSI. As for ROC-AUC, MaxViT is the highest score compared to the other encoders for both WSI and the ensemble, with the ROC-AUC improving to 0.852 for the ensemble. PR-AUC(WT) is the value when WT is treated as a positive class and IDH1 as a negative, and PR-AUC(IDH1) is the value when IDH1 is positive. For PR-AUC(WT), the highest result in WSI is 0.830 for CoAtNet, and the highest result in the ensemble is 0.868 for MaxViT. For PR-AUC(IDH1), the highest result in WSI is 0.819 for MaxViT, and the highest result in the ensemble is 0.833 for ResNet50(CLAM). In particular, MaxViT showed the best performance, achieving an accuracy of 0.778 and a ROC-AUC of 0.852. These scores are the highest in this work. Our results suggest that both WSI and clinical data can be used to predict IDH1 mutation status and that combining them can enhance the prediction performance. We consider that WSI-based and clinical data-based predictions complemented each other, leading to improved performance. Additionally, the ROC curves are shown in Fig. 5. We plotted the ROC curves for each fold, the averaged ROC curve, and the confidence band. The confidence band represents ± 1 standard deviation of the averaged ROC curve. Fig. 5(a–c) displays the ROC curves obtained from the WSI, clinical data, and the ensemble approach, respectively. For the ROC curve of the WSI, we used the MaxViT results with the highest AUC. As can be seen, the ensemble approach obtains better results than imaging (WSI) or clinical data-based methods alone.

### 3.3. Attention visualization of WSI

**Fig. 6.**
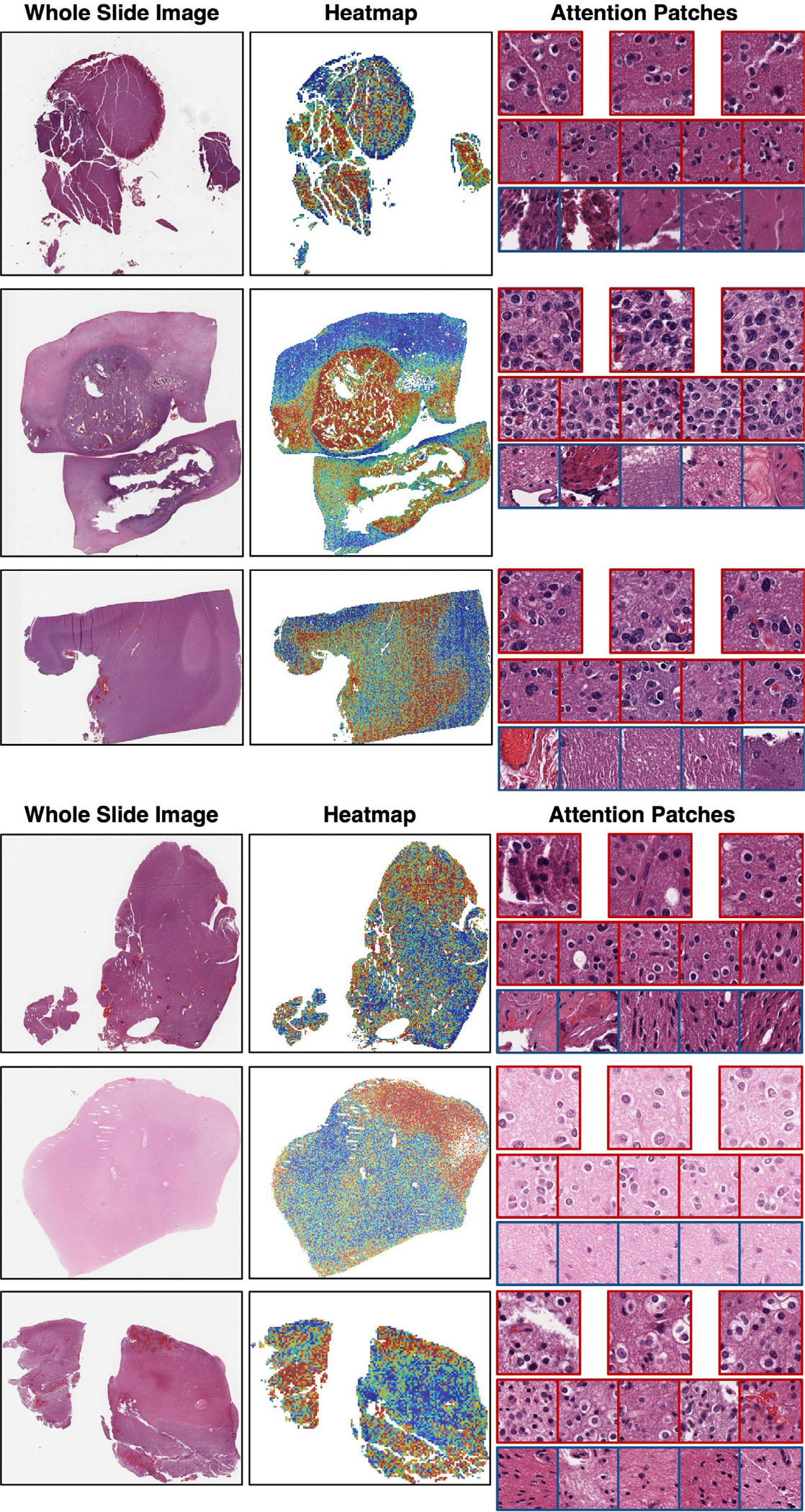
Example of attention heatmaps and attention patches (MaxViT) for WSIs. The regions with high attention scores are represented by red, and those with low scores are represented by blue. These three cases have positive predicted and ground truth labels for the IDH1 mutation. The histological features differ between high attention patches and low attention patches.

Fig. 6 illustrates a heatmap visualizing the attention score of ABMIL, employing MaxViT as the encoder for example WSIs. Fig. 6 displays cases with a positive IDH1 mutation, which ABMIL also correctly predicted as IDH1 positive. This visualization helps to identify regions of interest that contribute to the prediction without requiring patch-level annotations. It is expected to lead to interpreting morphological features associated with IDH1 mutation. Regions with high attention scores are colored in red, while those with low attention scores are colored in blue. Examples of high attention and low attention patches in the heatmap are shown in “Attention Patches” of Fig. 6. The high attention patches have “perinuclear halo” (fried egg appearance) that appears bright and clear around the cell nucleus. This “perinuclear halo” is a common morphological feature found in oligodendroglioma. In contrast, this feature was absent in the low attention patches. Thus, we see our approach can generate interpretable heatmaps. Therefore, it is expected to identify morphological features related to IDH1 gene mutation. In addition to the perinuclear halo, there may be other morphological features due to IDH1 mutation.

### 3.4. Interpretability of clinical data model

**Fig. 7.**
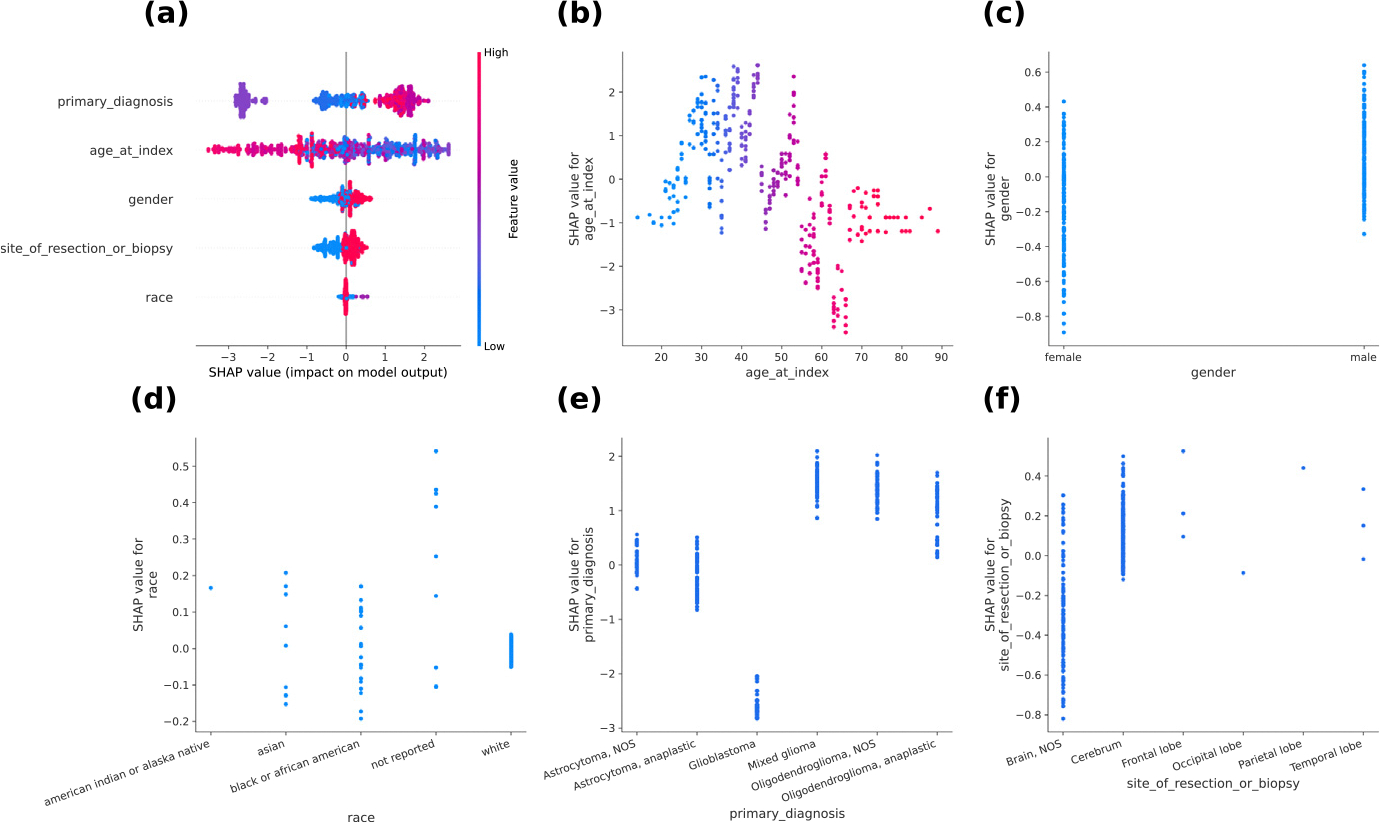
Feature importance with SHAP. (a): Beeswarm Plot. (b)–(f): Dependence Plot.

Finally, we examined the contribution of features using SHAP (SHapley Additive exPlanations) values. Fig. 7 shows the feature importance at fold 0 with SHAP. A higher SHAP value means a higher impact on the prediction of IDH1 mutation positive. The beeswarm plot illustrates which features affect the model’s output as shown in Fig. 7(a). It reveals that the primary diagnosis and age were particularly important, while gender, site of resection or biopsy, and race had lower importance. The dependence plot is a scatter plot between the feature value and its corresponding SHAP values in Fig. 7(b)–(f). For the age, we can see that the influence on IDH1 mutation prediction was higher for patient in their 20 s to 40 s and lower in those aged 50 or older. Indeed, it has been reported that IDH mutations are frequently encountered in patients under 55 years of age [35]. Therefore, age is an essential variable for predicting IDH1 mutation. For the primary diagnosis, the influence was particularly low for “Glioblastoma” as shown in Fig. 7(e). This is probably due to the low frequency of IDH1 mutation in glioblastoma patients. For the site of resection or biopsy, the influence on IDH1 mutation prediction of “Brain, NOS” was lower than other brain sites, as can be seen in Fig. 7(f). In other words, it is suggested that “Brain, NOS” contributes to wild-type prediction. Indeed, “Brain, NOS” tends to be more common in the wild-type (see Table 1). However, “Brain, NOS” means that the site of the brain is unknown, and it is a term used provisionally when the primary site cannot be identified clearly. Therefore, it may not be appropriate to conclude that “Brain, NOS” contributes to wild-type prediction since it does not have accurate information about the brain site like other categories. Furthermore, the site of resection or biopsy is correlated with the primary diagnosis (see Fig. 2). For these reasons, it seems that the site of resection or biopsy should not be used due to the fact the brain site is unclear and to avoid multicollinearity.

## 4. Conclusion

In this work, we proposed a method to predict glioma gene mutations by combining pathological images and clinical data. We focused on the IDH1 mutation, the most important factor in glioma diagnosis. The proposed approach trained separate models on pathological images and clinical data, and then the final prediction results were obtained by taking the average of their outputs. Our results suggest that the ensemble method achieved higher classification performance than the individual models. In addition, the results of feature importance analysis in the clinical data model, namely the LightGBM showed that primary diagnosis and age were the most important variables. Visualization of attention score in the pathological image model, namely the ABMIL suggested that the model can recognize histological features such as perinuclear halo. Despite the promising results, the ensemble model studied here currently does not take into account gene expression data that can feed further information to the framework. Adding further genomics information as further channels in a multichannel ensemble model and generalizing the proposed late fusion for discerning other gene mutations and studying their results constitutes are important future directions. Also, utilizing autoencoders [36] and other deep learning CNN and transformer models within the pathological imaging channel can be ensembled to maximize the information extracted from pathology domain.

## Figures and Tables

**Table 1:** Our dataset and clinical variables details in terms of IDH1 mutation versus wild-type (WT).

	IDH1	Wild-Type	Total

# of cases	321	225	546
# of WSIs	603	603	1206
Mean **Age** ± SD (years)	41.1 ± 12.2	54.2 ± 15.7	46.5 ± 15.2

**Gender**			
Male	186 (57.9%)	128 (56.9%)	314 (57.5%)
Female	135 (42.1%)	97 (43.1%)	232 (42.5%)

**Race**			
White	297 (92.5%)	202 (89.8%)	499 (91.4%)
Black or african american	12 (3.7%)	15 (6.7%)	27 (4.9%)
Asian	5 (1.6%)	4 (1.8%)	9 (1.6%)
American indian or alaska native	0 (0%)	1 (0.4%)	1 (0.2%)
Not reported	7 (2.2%)	3 (1.3%)	10 (1.8%)

**Primary diagnosis**			
Astrocytoma, anaplastic	63 (19.6%)	50 (22.2%)	113 (20.7%)
Astrocytoma, NOS	39 (12.1%)	13 (5.8%)	52 (9.5%)
Oligodendroglioma, anaplastic	48 (15%)	18 (8%)	66 (12.1%)
Oligodendroglioma, NOS	78 (24.3%)	20 (8.9%)	98 (17.9%)
Glioblastoma	4 (1.2%)	100 (44.4%)	104 (19%)
Mixed glioma	89 (27.7%)	24 (10.7%)	113 (20.7%)

**Site of resection or biopsy**			
Cerebrum	265 (82.6%)	109 (48.4%)	374 (68.5%)
Brain, NOS	46 (14.3%)	114 (50.7%)	160 (29.3%)
Temporal lobe	5 (1.6%)	0 (0%)	5 (0.9%)
Frontal lobe	3 (0.9%)	2 (0.9%)	5 (0.9%)
Occipital lobe	1 (0.3%)	0 (0%)	1 (0.2%)
Parietal lobe	1 (0.3%)	0 (0%)	1 (0.2%)

WSI — whole slide image, SD — standard deviation, NOS — not otherwise specified.

**Table 2:** ABMIL hyperparameters optimized for each encoder.

Hyperparameter	VGG16	InceptionV3	ResNet50	EfficientNetV2 B0

Batch size	1	1	1	1
Loss	CrossEntropy	CrossEntropy	CrossEntropy	CrossEntropy
Epochs	100	100	100	100
Early stopping patience	20	20	–	–
GatedAttention Hidden layer	4096-512-256-1	2048-512-256-1	2048-512-256-1	1280-512-256-1
Dropout rate	0.25	0.25	0.25	0.25
Optimizer	SGD	SGD	SGD	SGD
Weight decay	3.0e–7	1.6e–6	1.6e–6	1.6e–6
Scheduler	CosineLRScheduler	CosineLRScheduler	CosineLRScheduler	CosineLRScheduler
Peak lr	3.0e–5	3.0e–4	3.0e–4	3.0e–4
Min lr	2.0e–8	1.5e–8	1.5e–8	1.5e–8
Warmup epochs	8	12	12	12
Warmup lr init	5.4e–8	7.6e–8	7.6e–8	7.6e–8

Hyperparameters	EfficientNetV2 B1	SwinTransformerV2	CoAtNet	MaxViT

Batch size	1	1	1	1
Loss	CrossEntropy	CrossEntropy	CrossEntropy	CrossEntropy
Epochs	100	100	100	100
Early stopping patience	20	20	20	20
GatedAttention Hidden layer	1280-512-256-1	768-512-256-1	768-512-256-1	512-512-256-1
Dropout rate	0.25	0.25	0.25	0.25
Optimizer	SGD	SGD	SGD	SGD
Weight decay	1.6e–6	1.2e–7	3.0e–7	1.6e–6
Scheduler	CosineLRScheduler	CosineAnnealingLR	CosineAnnealingLR	CosineLRScheduler
Peak lr	3.0e–4	1.6e–4	3.0e–5	3.0e–4
Min lr	1.5e–8	8.7e–7	1.0e–7	1.5e–8
Warmup epochs	12	–	–	12
Warmup lr init	7.6e–8	–	–	7.6e–8

**Table 3:** Optimized LightGBM hyperparameters.

Hyperparameter	LightGBM

objective	binary
boosting_type	gbdt
importance_type	split
learning_rate	0.08171120044551923
reg_alpha	1.2405777881849258e-08
reg_lambda	1.5937337120820927
num_leaves	27
max_depth	−1
colsample_bytree	0.6215618710838064
subsample	0.4438242926449302
subsample_freq	2
subsample_for_bin	200 000
min_child_samples	26
min_child_weight	0.001
min_split_gain	0.0
n_estimators	100
num_iterations	268

**Table 4:** Experimental Results. The 10-fold average performance is shown with ± SD.

Models	#Params	Accuracy	Precision	Recall	F1-score	ROC-AUC	PR-AUC(WT)	PR-AUC(IDH1)

Clinical								
LightGBM [27]	-	0.719 ± 0.073	0.715 ± 0.087	0.774 ± 0.070	0.741 ± 0.066	0.782 ± 0.076	0.818 ± 0.056	0.759 ± 0.082

WSI								
VGG16 [28]	134.26M	0.700 ± 0.059	0.698 ± 0.088	0.747 ± 0.057	0.720 ± 0.064	0.780 ± 0.063	0.777 ± 0.064	0.771 ± 0.083
InceptionV3 [29]	21.79M	0.735 ± 0.059	0.743 ± 0.088	0.775 ± 0.057	0.752 ± 0.064	0.798 ± 0.063	0.808 ± 0.064	0.777 ± 0.083
ResNet50 [30] (ABMIL)	23.51M	0.733 ± 0.048	0.738 ± 0.074	0.753 ± 0.058	0.743 ± 0.058	0.805 ± 0.048	0.805 ± 0.046	0.789 ± 0.077
EfficientNetV2 B0 [31]	5.86M	0.736 ± 0.049	0.744 ± 0.073	0.758 ± 0.070	0.747 ± 0.053	0.799 ± 0.052	0.811 ± 0.044	0.769 ± 0.082
EfficientNetV2 B1 [31]	6.86M	0.743 ± 0.074	0.750 ± 0.087	0.762 ± 0.108	0.751 ± 0.084	0.803 ± 0.064	0.818 ± 0.045	0.772 ± 0.098
SwinTransformerV2 [32]	27.58M	0.730 ± 0.065	0.739 ± 0.088	0.736 ± 0.097	0.734 ± 0.084	0.799 ± 0.059	0.800 ± 0.049	0.784 ± 0.082
CoAtNet [33]	40.96M	**0.752 ± 0.044**	**0.765 ± 0.082**	0.769 ± 0.077	**0.761 ± 0.048**	0.821 ± 0.054	**0.830 ± 0.046**	0.802 ± 0.082
MaxViT [34]	28.64M	0.739 ± 0.051	0.743 ± 0.066	0.757 ± 0.111	0.745 ± 0.070	**0.823 ± 0.049**	0.821 ± 0.050	**0.819 ± 0.068**
ResNet50 (CLAM) [25]	8.54M	0.725 ± 0.080	0.710 ± 0.098	**0.819 ± 0.076**	0.755 ± 0.068	0.806 ± 0.063	0.810 ± 0.047	0.780 ± 0.095

Ensemble								
VGG16	134.26M	0.749 ± 0.067	0.727 ± 0.084	0.834 ± 0.057	0.775 ± 0.062	0.820 ± 0.068	0.841 ± 0.056	0.800 ± 0.096
InceptionV3	21.79M	0.768 ± 0.067	0.750 ± 0.084	0.845 ± 0.057	0.791 ± 0.062	0.835 ± 0.068	0.855 ± 0.056	0.814 ± 0.096
ResNet50 (ABMIL)	23.51M	0.777 ± 0.054	0.751 ± 0.071	**0.859 ± 0.045**	**0.800 ± 0.049**	0.844 ± 0.059	0.860 ± 0.052	0.832 ± 0.078
EfficientNetV2 B0	5.86M	0.767 ± 0.060	0.752 ± 0.082	0.831 ± 0.054	0.787 ± 0.055	0.839 ± 0.063	0.857 ± 0.055	0.824 ± 0.092
EfficientNetV2 B1	6.86M	0.773 ± 0.056	0.761 ± 0.082	0.831 ± 0.046	0.791 ± 0.050	0.842 ± 0.066	0.863 ± 0.055	0.818 ± 0.100
SwinTransformerV2	27.58M	0.767 ± 0.061	0.745 ± 0.081	0.845 ± 0.045	0.790 ± 0.056	0.839 ± 0.064	0.856 ± 0.053	0.829 ± 0.085
CoAtNet	40.96M	0.766 ± 0.073	0.753 ± 0.086	0.824 ± 0.069	0.784 ± 0.068	0.846 ± 0.067	0.866 ± 0.056	0.828 ± 0.086
MaxViT	28.64M	**0.778 ± 0.062**	**0.765 ± 0.075**	0.827 ± 0.088	0.792 ± 0.065	**0.852 ± 0.059**	**0.868 ± 0.050**	0.831 ± 0.080
ResNet50 (CLAM)	8.54M	0.771 ± 0.056	0.745 ± 0.076	0.857 ± 0.070	0.794 ± 0.056	0.843 ± 0.057	0.857 ± 0.050	**0.833 ± 0.081**
